# α4-integrins control viral meningoencephalitis through differential recruitment of T helper cell subsets

**DOI:** 10.1186/2051-5960-2-27

**Published:** 2014-03-07

**Authors:** Veit Rothhammer, Andreas Muschaweckh, Georg Gasteiger, Franziska Petermann, Sylvia Heink, Dirk H Busch, Mathias Heikenwälder, Bernhard Hemmer, Ingo Drexler, Thomas Korn

**Affiliations:** 1 Klinikum rechts der Isar, Department of Neurology, Technische Universität München, Ismaninger Str. 22, 81675 Munich, Germany; 2 Institute of Virology, Helmholtz Zentrum München, Technische Universität München, Schneckenburger Str. 8, 81675 Munich, Germany; 3 Howard Hughes Medical Institute and Immunology Program, Memorial Sloan-Kettering Cancer Center, New York, NY 10065, USA; 4 Institute for Medical Microbiology, Immunology, and Hygiene, Technische Universität München, Trogerstr. 30, 81675 Munich, Germany; 5 Munich Cluster for Systems Neurology (SyNergy), Munich, Germany; 6 Institute for Virology, Düsseldorf University Hospital, Heinrich-Heine-University, Universitätsstr. 1, 40225 Düsseldorf, Germany

## Abstract

**Introduction:**

Natalizumab blocks α4-integrins and is a prototypic agent for a series of anti-inflammatory drugs that impair trafficking of immune cells into the CNS. However, modulation of the access of immune cells to the CNS is associated with impaired immune surveillance and detrimental viral infections of the CNS. Here, we explored the potency of cellular immune responses within the CNS to protect against viral encephalitis in mice with T cell conditional disruption of VLA-4 integrin (α4β1) expression.

**Results:**

While VLA-4 expression in virus specific Th1 cells is non-redundant for their ability to access the CNS, α4-integrin deficient Th17 cells enter the CNS compartment and generate an inflammatory milieu upon intrathecal vaccinia virus (VV) infection. However, in contrast to Th1 cells that can adopt direct cytotoxic properties, Th17 cells fail to clear the virus due to insufficient Eomes induced perforin-1 expression.

**Conclusion:**

The quality of the intrathecal cellular antiviral response under conditions of impaired VLA-4 function jeopardizes host protection. Our functional *in vivo* data extend our mechanistic understanding of anti-viral immunity in the CNS and help to estimate the risk potential of upcoming therapeutic agents that target the trafficking of immune cells into distinct anatomical compartments.

## Introduction

Autoimmune inflammation of the CNS in multiple sclerosis (MS) and its animal model, experimental autoimmune encephalomyelitis (EAE), is mediated by antigen specific Th1 and Th17 cells [1]. For many years integrin targeted blocking of T helper cell trafficking into the CNS has appeared to be an attractive approach to treat immunopathology in MS [2]. In particular, monoclonal antibodies (natalizumab) to the α4 subunit of the integrin VLA-4 (α4β1 heterodimer) were successfully used to prevent the influx of immune cells into the CNS and to treat CNS autoimmunity [3]. However, in experimental models it has been shown that distinct encephalitogenic T cell subsets vary in their equipment with VLA-4 [4]. While Th1 cells maintain high amounts of VLA-4 expression, Th17 cells are low in VLA-4. As a consequence, blockade of VLA-4 is more efficient in preventing the recruitment of Th1 cells than of Th17 cells into the CNS parenchyma.

Although considered as an “immune privileged” organ, the CNS is still patrolled by T cells as a means of immune surveillance [5]. The contribution of CD4^+^ vs CD8^+^ effector memory T cells in the migratory and resident pools of lymphocytes specific to a given pathogen has been investigated in skin infection but is unclear in the CNS [6]. In the treatment of organ specific autoimmunity and chronic inflammation, efforts are increasing to market compounds that either inhibit immune cell trafficking [7-10] or cytokine networks that affect distinct T helper cell subsets in a differential manner (anti-IL-23p19, anti-IL-17A [11,12], anti-GM-CSF (NCT01517282), anti-IL-6R [13]). However, preclinical models to investigate niche specific immune surveillance and host defense in the CNS are rare. Indeed, efalizumab, a blocking antibody to the integrin αL was withdrawn from the market in 2009 because of viral meningitis and cases of JC virus induced progressive multifocal leukencephalopathy (PML) [14].

Here, we established a CNS specific viral infection model that allowed us to analyze the contribution of distinct T helper cell subsets to host protection. We chose vaccinia virus (VV) infection where the importance of virus specific T helper cell responses has been analyzed previously [15,16]. Vaccinated mice were found to be protected from intrathecal (i.th.) infection with VV due to cellular immunity. In the absence of CD8^+^ T cells, Th1 like cells were sufficient to protect mice from intrathecal VV infection. Access of Th1 cells into the infected CNS compartment was dependent on VLA-4 expression. Although virus specific Th17 cells were able to migrate into the CNS in the absence of VLA-4, CNS recruited and infected macrophages were not cleared by Th17 cells since Th17 cells – in contrast to Th1 cells – were deficient in perforin-1 expression. These data highlight a dominant role of Th1 cells in antiviral tissue-specific immunity. Our data further suggest that as in autoimmune inflammation of the CNS, virus specific Th1 cells are dependent on VLA-4 to enter into the CNS and virus infection does not overcome the requirement for Th1 cells to express VLA-4. Thus, integrin targeted therapeutic interventions in autoimmunity and chronic inflammation need to be refined in order to not jeopardize organ specific immune surveillance and host protection.

## Materials and methods

### Animals, immunization, and infection

*Foxp3gfp*.KI mice [17,18] and *Itga4*^*flox/flox*^ mice [19] have been described previously. *CD4 Cre* mice, *Ifng*^*-/-*^ mice, *Rag1*^*-/-*^ mice, *Prf1*^*-/-*^ mice, and wild type C57BL/6 mice were obtained from Jackson Laboratories. CD45.1^+^ OT-II mice were kindly provided by DH Busch (Institute for Medical Microbiology, Immunology, and Hygiene, Technische Universität München). All mouse strains were on pure C57BL/6 background.

Mice were immunized by subcutaneous injection of 100 μl of an emulsion of 1 × 10^8^ IU MVA or PBS in complete Freund’s adjuvant (CFA). For *in vivo* blockade of IFN-γ, mice were treated with every other day i.p. injections of a neutralizing antibody to IFN-γ (R4-6A2, BioXCell, West Lebanon, USA; 200 μg) or isotype control starting on day 9 after immunization. In a similar regimen, blocking antibodies to integrin α4 (PS/2, BioXCell, West Lebanon, USA; 200 μg), depleting antibodies to CD8 (YTS169.4, BioXcell; 200 μg) or CD4 (GK1.5, BioXcell; 200 μg) were administered every other day from day 9 or day 10 after immunization, respectively.

Intrathecal infection was performed as previously described [20]. In brief, VV was inoculated into the cisterna magna of mice in deep anaesthesia by means of transcutaneous suboccipital puncture. Clinical signs of disease as well as weight loss in percent of initial weight (means + SEM) were monitored daily.

For adoptive transfer experiments, naïve T cells were isolated by magnetic sorting (CD4^+^CD62L^+^; T cell isolation kit II, mouse; Miltenyi Biotec, Germany) from CD45.1^+^ OT-II mice and differentiated *in vitro* into Th1 or Th17 cells. The differentiation status was checked on day 4 by intracellular cytokine staining and 2 × 10^6^ cytokine positive T cells were injected i.v. into *Rag1*^*-/-*^ recipient mice, which had been infected with VV-Ova one day prior to T cell transfer.

Animals were kept in a specific pathogen-free facility at the Technische Universität München. All experimental protocols were approved by the standing committee for experimentation with laboratory animals of the Bavarian state authorities (“Governmental Department of Upper Bavaria, Approved animal experimental proposals No 55.2-1-54-2531-88-08 and No 55.2.1-54-2532-29-13 according to §8.1, German law for experimentation with laboratory animals”) and carried out in accordance with the corresponding guidelines.

### Virus strains

Replication competent VV Western Reserve strain (VV) was provided by B Moss (National Institutes of Health, Bethesda, MD). Recombinant viruses encoding enhanced green fluorescent protein (VV eGFP) or full-length ovalbumin (VV-Ova) based on the Western Reserve strain were provided by JW Jewdell and JR Bennink (National Institutes of Health, Bethesda, MD). VV and replication deficient Modified Vaccinia Virus Ankara strain (MVA, cloned isolate IInew) used in this study were propagated and titered according to standard methodology [21].

### T cell differentiation

Naïve T cells (CD4^+^CD62L^high^CD25^-^) were isolated from lymph nodes and spleen by magnetic sorting (T cell isolation kit II, mouse; Miltenyi Biotec, Germany). Purity was in general higher than 95% as controlled by FACS staining. Naïve T cells were stimulated for 3 to 5 days with plate-bound antibody to CD3 (145-2C11, 4 μg/ml) and antibody to CD28 (PV-1, 2 μg/ml). Recombinant cytokines were added to the differentiation cultures as indicated: human TGF-β1 (2 ng/ml) and mouse IL-6 (50 ng/ml) for Th17, mouse IL-12 (10 ng/ml) and anti-IL-4 (10 μg/ml) for Th1, all R&D Systems.

### Preparation of splenic and CNS mononuclear cells and antibody staining

Mononuclear cells were isolated from either spleen or CNS at the peak of disease (d4-d5 after intrathecal challenge). After perfusion through the left cardiac ventricle with cold PBS, the brain including cerebellum was dissected and the spinal cord flushed out with PBS by hydrostatic pressure. CNS tissue was digested with collagenase D (2.5 mg/ml, Roche Diagnostics, Indianapolis IN) and DNAseI (1 mg/ml, Sigma, Saint Louis, MO) at 37°C for 45 min. Mononuclear cells were isolated by passing the tissue through a cell strainer (70 μm) and percoll gradient (37% over 70%) centrifugation. Mononuclear cells were removed from the interphase, washed and resuspended in culture medium for further analysis. For isolation of mononuclear cells from spleen, spleens were mashed through a cell strainer (70 μm) and red blood cells were removed using BD Pharm Lyse (BD Biosciences). Surface staining of T cells was carried out with antibodies to CD3 (14-2C11), CD4 (RM4-5), CD8 (53-6.7), CD11b (M1/70), CD25 (PC61 or 7D4), CD44 (IM7), CD45 (30-F11) and Nk1.1 (PK136). All antibodies were purchased from BD Biosciences. Fluorescence-labeled MHC class I H-2K^b^/B8R_20-27_ (TSYKFESV) multimers were provided by DH Busch.

### Intracellular cytokine staining

Cells were stimulated in culture medium containing phorbol 12-myristate 13-acetate (PMA, 50 ng/ml, Sigma), ionomycin (1 μg/ml, Sigma), and monensin (GolgiStop 1 μl/ml, BD Biosciences) at 37°C and 10% CO_2_ for 4 hours. After staining of surface markers, cells were fixed and permeabilized (Cytofix/Cytoperm and Perm/Wash buffer, BD Biosciences) followed by staining with monoclonal antibodies to mouse IL-2, IL-17, or IFN-γ (BD Biosciences) and flow cytometric analysis (CYAN, Beckmann/Coulter).

### Histologic analysis

For detection of VV-infected cells or macrophages, paraformaldehyde (PFA) (4%) fixed and paraffin embedded CNS tissue sections were incubated with Bond Primary Antibody Diluent (Leica) containing either polyclonal rabbit anti-VV serum (1:1000; Quartett Immunodiagnostika & Vertriebs-GmbH, Berlin) or monoclonal antibodies against Mac-3 (1:750; M3/84) purchased from BD Pharmingen. IHC staining was performed on an automated Leica BOND-MAX instrument using Bond Polymer Refine Detection Solution for DAB. For detection of GFAP, PFA-fixed and paraffin-embedded CNS sections were incubated with Dako polyclonal rabbit anti-GFAP antibodies (Z0034; 1:13000) in Ventana buffer and staining was performed on a Ventana NexES IHC Slide Stainer using iVIEW DAB Detection Kit (Ventana). Images were taken using the Leica SCN400 slide scanner analysis software or were acquired on an Olympus BX53 Microscope (DP72 camera) using the cellSens 1.8 digital imaging software (Olympus).

### Quantitative PCR analysis

For quantitative PCR, RNA was extracted from magnetic bead-purified or flow cytometry-sorted cells *ex vivo* or after *in vitro* differentiation using RNeasy columns (Qiagen, Valencia, CA). Complementary DNA was transcribed as recommended (Applied Biosystems, Foster City, CA) and used as template for quantitative PCR. Primer plus probe mixtures were obtained from Applied Biosystems. The Taqman analysis was performed on a StepOne system from Applied Biosystems. The gene expression was normalized to the expression of β-actin.

### Western blotting

T cells were lysed and denatured using RiPA buffer (Sigma-Aldrich). The protein lysates were separated by SDS-PAGE in 4–12% NuPAGE Bis-Tris Mini gels and transferred to nitrocellulose membranes (Invitrogen). After blocking with 5% low-fat dry milk in TBS-T, membranes were incubated with primary antibodies to Prf-1 (ab7203, Abcam) and β-actin (Abcam) in blocking solution overnight at 4°C. Primary antibody binding was detected with HRP-conjugated secondary antibodies (Dianova). The signal was visualized by enhanced chemiluminescence (Novex ECL, Invitrogen).

### Antigen specific proliferative and cytokine responses

For CD154 (CD40L) staining, spleens from MVA immunized *CD4 Cre*^*-*^ × *Itga4*^*flox/flox*^ or *CD4 Cre*^*+*^ × *Itga4*^*flox/flox*^ mice were dissected on day 10 after immunization. Single cell suspensions were prepared and cells were seeded on a 12 well flat-bottom plate at a density of 2 × 10^6^ cells/well. Single cells were restimulated with a mixture of I-A^b^-restricted VV peptides (A33R, B2R, B5R, L4R; 30 μg/ml each) for 6 hours in the presence of brefeldin A (5 μg/ml) during the last 3 hours of incubation followed by surface and intracellular staining for CD40L (CD154 (MR1), eBioscience) and cytokines as indicated. A33R_116-130_ (YQLFSDAKANCTAES), B2R_46-60_ (VKDKYMWCYSQVNKR), B5R_46-60_ (FTCDQGYHSSDPNAV) and L4R_176-190_ (ISKYAGINILNVYSP) were obtained from Biosyntan, Berlin.

### Retroviral transduction of T cells

pMIG Eomes (GFP) and control pMIG (GFP) retroviral constructs were a kind gift from SL Reiner (University of Pennsylvania, Philadelphia, PA) and FJ Quintana (Harvard Medical School, Boston, MA). Phoenix-Eco cells (PMID: 18432682, a kind gift of H-M Jaeck, Erlangen) were transiently transfected with plasmids by calcium phosphate precipitation in the presence of 25 μM chloroquine (Sigma Aldrich). Retroviral supernatants were collected two days post transfection. T cells were transduced 24 hours post polyclonal *in vitro* activation of naïve sorted CD4^+^ T cells with TGF-β and IL-6 (Th17). Retroviral supernatant and 4 μg/ml polybrene (Merck Millipore) were added to the pre-committed Th17 cells and a spin transduction was performed (2000 rpm, RT, 1 hour). Cells were further cultured for 3 days in Th17 polarizing conditions before GFP expressing cells were purified by FACS sorting and subjected to quantitative PCR analysis.

### Plaque assay

CNS tissue was digested as described and single cell suspensions were obtained by passing digested CNS tissue through a 70-μm nylon filter. Pelleted cells were subjected to three freeze-thaw cycles (-80°C and 37°C) and sonicated three times for 1 min. Serial dilutions in RPMI-1640 medium containing 10% FCS were added in duplicates to 90% confluent RK-13 cells seeded in 6-well plates and incubated for 24 hours at 37°C. Plaques were counted after crystal violet staining.

### Neutralization assay

Mouse serum samples were collected at day 10 post immunization (MVA/CFA or PBS/CFA). All serum samples were heat-inactivated at 56°C for 30 min prior to testing. Serum dilutions were incubated with 10,000 PFU of sucrose gradient purified VV eGFP for 1 hour at 37°C and 5% CO_2_ in a 96-well plate. RK-13 cells were added and infection (MOI = 0.1) was carried out over night at 37°C and 5% CO_2_. Cells were harvested, washed and fixed in 1% paraformaldehyde. Percentage of VV-infected cells was determined by measuring eGFP expression in flow cytometric analyses (CYAN, Beckmann/Coulter). The percentage of virus neutralization was defined as (1-[percentage of GFP-expressing cells]/[percentage of GFP-expressing cells in controls without serum]) × 100.

### Statistical analysis

Statistical evaluations of cell frequency measurements and gene expression levels were performed with the unpaired Student’s t test. Two-tailed p values < 0.05 were considered significant. Weight scores are given as means ± SEM. Multiple comparisons were performed by two-way-ANOVA and Bonferroni post-testing.

## Results

### Intrathecal vaccinia virus (VV) infection causes lethal encephalitis

**Figure 1:**
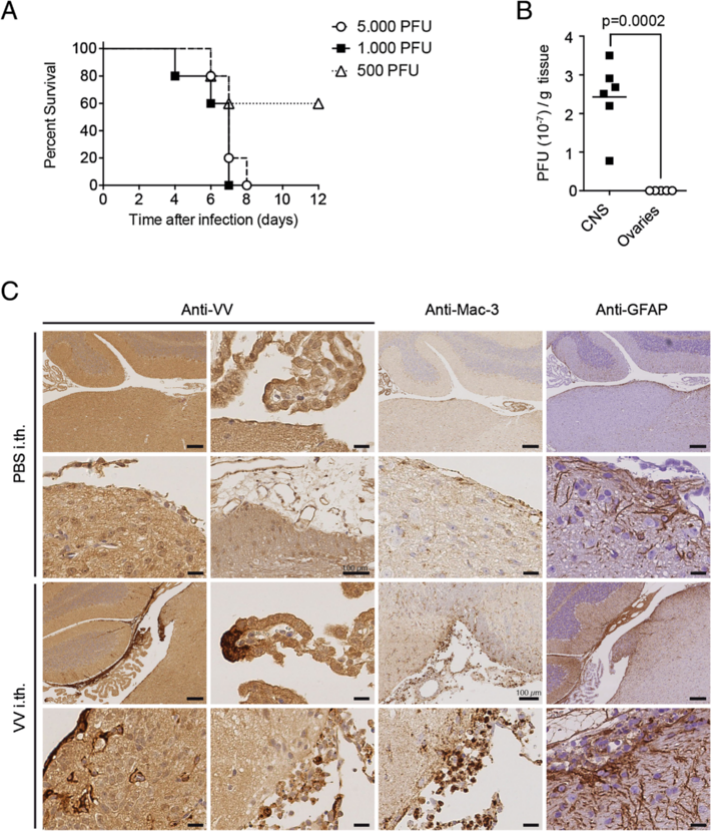
**Intrathecal infection with VV causes lethal encephalitis. (A)** C57BL/6 wild type mice were injected intrathecally by suboccipital puncture of the cisterna magna with various doses of VV. Percentage of surviving mice are depicted in Kaplan-Meier curves (n = 5 per group). **(B)** Virus loads in CNS and ovaries of wild type mice infected intrathecally with VV were measured on day 5 after infection (Student’s t test, n ≥ 5). **(C)** Wild type mice injected with VV were subjected to histologic analyses on day 5 after infection. Unless otherwise indicated, scale bars represent 200 μm in the low magnification and 20 μm in the higher magnification photomicrographs.

To establish a model of viral encephalitis, female C57BL/6 mice were injected intrathecally with VV by suboccipital puncture of the cisterna magna. By titrating the dose of the inoculum, the half lethal dose (LD50) was determined to be approximately 500 plaque forming units (PFU) (Figure 1A). In systemic infection, VV is known to preferentially replicate in the ovaries upon hematogenous dissemination. We therefore measured viral loads in CNS and ovaries of mice injected i.th. with 1.000 PFUs of VV. While high titers of VV were measured in the CNS, virus was not detected in the ovaries of intrathecally infected mice (Figure 1B) indicating that intrathecal injection of VV led to a compartmentalized infection within the CNS without systemic dissemination. Within the CNS compartment, VV antigen was detected in ventricular lining cells and plexus epithelium cells as well as in astrocytes of the glia limitans. Some innate immune cells of monocytic origin that were recruited to the infected CNS compartment were also positive for VV antigen (Figure 1C).

**Figure 2:**
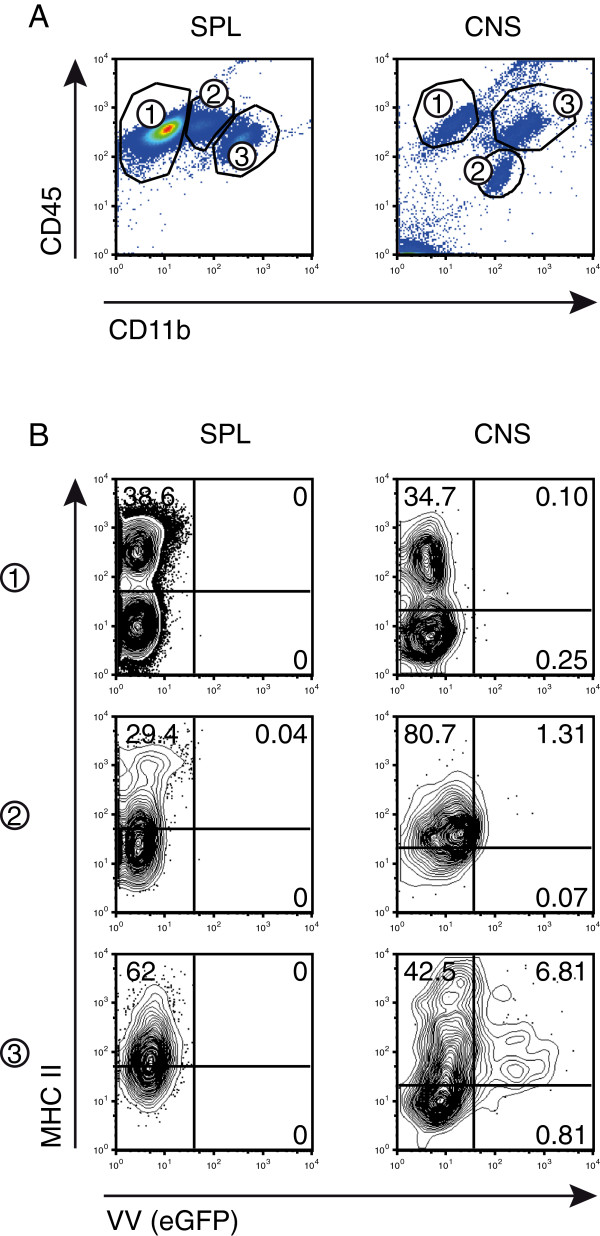
**MHC class II positive macrophages are targets of VV infection in the CNS.** Wild type mice were infected intrathecally with 1.000 PFUs of VV (eGFP), a replication competent recombinant VV expressing enhanced green fluorescent protein (eGFP) in infected cells. On day 4 after infection, mice were sacrificed, and spleen and CNS mononuclear cells were isolated and analyzed by flow cytometry. In the live cell gate, the following populations were defined based on their expression of CD11b and CD45: **(A)** spleen (SPL), left panel: (1) CD11b^low^CD45^high^ lymphocytes, (2) CD11b^int^CD45^high^ dendritic cells, (3) CD11b^high^CD45^high^ macrophages. **(A)** CNS, right panel: (1) CD11b^low^CD45^high^ lymphocytes, (2) CD11b^int^CD45^int^ microglia, (3) CD11b^high^CD45^high^ macrophages. **(B)** These populations were analyzed as to their expression of eGFP and MHC class II by flow cytometry. Numbers indicate percentages of positive cells in each quadrant or gate (representative out of 3 independent experiments).

In order to determine immune cell targets of VV within the CNS, we infected wild type mice with recombinant VV expressing enhanced green fluorescent protein (VV eGFP). When analyzing eGFP expression in distinct cell populations of the CNS on day 4 after infection, we found a sizable fraction of eGFP expressing cells only within the CD11b^high^CD45^high^ macrophage compartment (Figure 2). This is in line with a recent study which identified infiltrating CD11b^+^CD45^+^ inflammatory monocytes as the predominant VV infected leukocyte population during VV skin infection [22]. Microglial cells (CD11b^+^CD45^low^), T cells and B cells did not show eGFP expression. In addition, we were unable to detect eGFP expressing cells in the spleens of intrathecally injected mice, further demonstrating that VV infection was restricted to the CNS compartment. Importantly, infected CD11b^+^CD45^high^ macrophages expressed MHC class II (Figure 2B). Thus, phagocytic cells were recruited from the systemic compartment in response to i.th. VV infection and became targets of virus replication but were unable to control the infection (see Figure 1A).

### Immunization with modified vaccinia virus (MVA) protects against VV encephalitis

**Figure 3:**
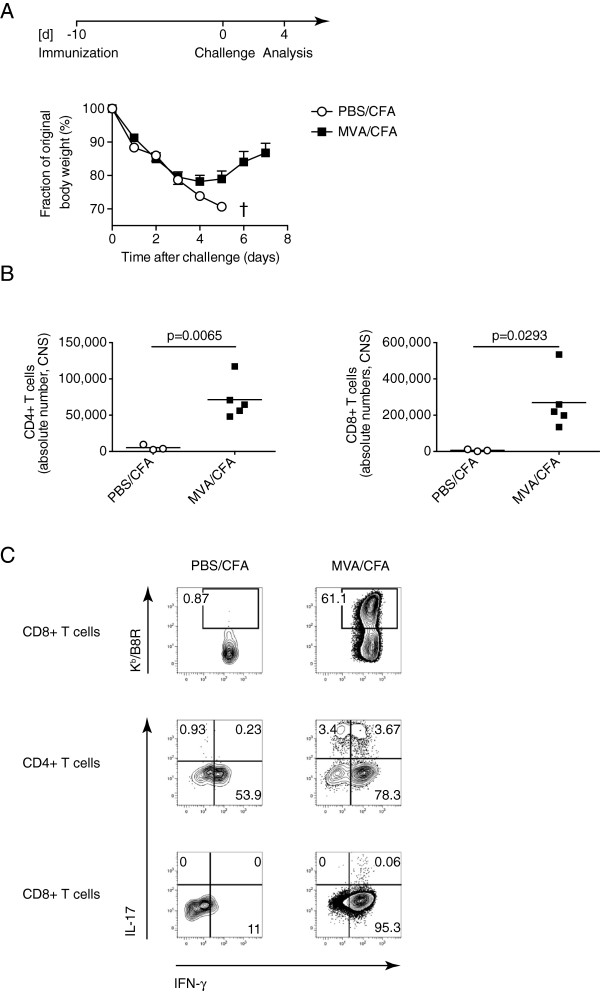
**Immunization with modified VV Ankara (MVA) confers protective immunity against viral encephalitis.** Wild type mice were immunized subcutaneously with a replication deficient strain of VV (Modified Vaccinia Virus Ankara, MVA) or PBS emulsified in complete Freund’s Adjuvant (CFA). On day 10 after immunization, mice were challenged intrathecally with VV, and weight courses were monitored daily as percentage of initial body weight (**A**, n = 5 mice per group). On day 4 after infection, CNS infiltrating mononuclear cells were isolated. **(B)** Absolute numbers of CNS infiltrating CD4^+^ and CD8^+^ T cells. Horizontal bars indicate means (Student’s t test; n ≥ 5 per group). **(C)** CNS derived T cells were analyzed as to their specificity to the VV MHC class I restricted epitope B8R by multimer staining and to their IL-17 and IFN-γ cytokine status by intracellular cytokine staining in the CD3^+^CD8^+^ CTL and CD3^+^CD4^+^ T helper cell compartment, respectively. Numbers indicate percentages of cells in the depicted gates or quadrants; representative out of more than 5 independent experiments).

As macrophages became infected during VV encephalitis but failed to eliminate the virus, we hypothesized that adaptive cellular immune responses were required for virus control. In order to investigate the contribution of antigen specific effector T helper cells to host defense against i.th. VV infection, we immunized wild type mice with a replication deficient strain of VV (MVA) emulsified in complete Freund’s adjuvant (CFA) 10 days prior to i.th. challenge with VV. CFA induces both Th1 and Th17 responses. Here, we were interested in investigating the role of these T helper cell subsets in the trade-off between host protection and immunopathology during infectious encephalitis. While sham-immunized mice died at day 6 after infection and largely failed to recruit either CD4^+^ T helper cells or VV-specific CTLs into the CNS compartment (Figure 3A,B), MVA immunized animals harbored both antigen specific CTLs and CD4^+^ T cells in the CNS and recovered from VV encephalitis (Figure 3A-C). While CTLs re-isolated from the CNS largely produced IFN-γ, the intrathecal CD4^+^ effector T cell compartment comprised IFN-γ producers, IL-17 producers, and IFN-γ/IL-17 double producers (Figure 3C). In summary, protective immunity against intrathecal VV infection was associated with the mobilization of both CTLs and CD4^+^ effector T helper cells to the CNS.

### Antibody mediated blockade of α4-integrins leads to relative enrichment of Th17 cells in the CNS

**Figure 4:**
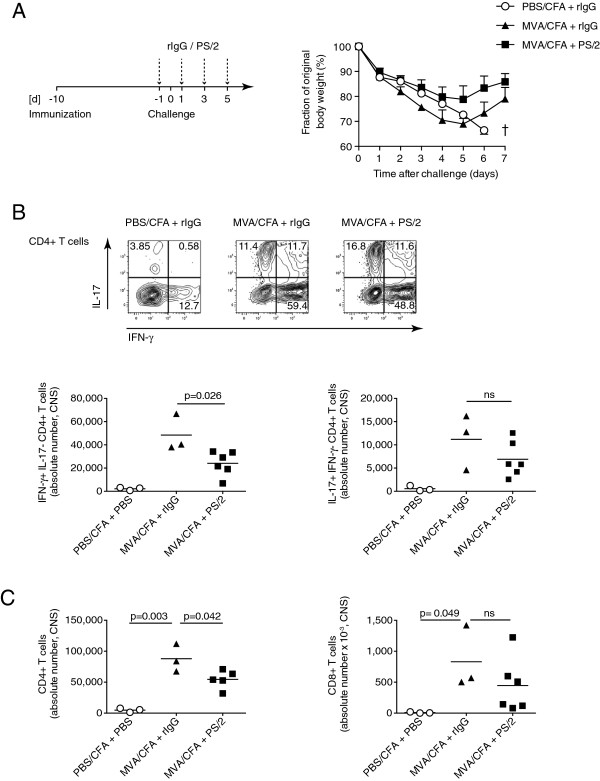
**Antibody mediated blockade of integrin α4 prevents entry of Th1, but not Th17 cells or CD8**^**+**^**T cells into the CNS.** Wild type mice were sham immunized (PBS/CFA) or immunized with MVA/CFA followed by intrathecal VV challenge on day 10 after immunization. Control IgG or antibodies to integrin α4 (PS/2) were administered every other day starting one day prior to VV challenge. **(A)** Weight courses of VV challenged animals (n = 5 per group). **(B, C)** On day 4 after infection, CNS infiltrating mononuclear cells were isolated and the absolute number of CD4^+^ T helper cells and CD8^+^ CTLs as well as the cytokine phenotype of CD4^+^ T helper cells from the CNS were assessed based on intracellular staining for IFN-γ and IL-17. (B; numbers indicate percentages of CD4^+^ T cells in each quadrant; representative out of 5 independent experiments. The absolute numbers of T cells were compared by Student’s t test, n ≥ 3).

We next wished to dissect the specific contribution of different T cell subsets to the protective effects of vaccination. Here, we took advantage of the differential integrin requirement for the recruitment of Th1 cells vs Th17 cells into the CNS compartment. In the EAE model, Th1 cells depend on α4-integrins in order to enter into the CNS during autoimmune inflammation. In contrast, Th17 cells are able to access the CNS compartment independently of α4-integrins using an LFA-1 dependent mechanism [4]. Here, we treated our MVA immunized mice with blocking antibodies to integrin α4 (PS/2) prior to i.th. VV challenge (Figure 4A). Notably, MVA immune mice survived i.th. VV infection in spite of integrin α4 blockade (Figure 4A). As expected, we found a relative abundance of IL-17 producing T cells as compared with IFN-γ positive T helper cells (Figure 4B). This finding supported the idea that – similar to T cell recruitment in autoimmune inflammation of the CNS – Th1 cells and Th17 cells exhibited distinct integrin requirements for their entry into CNS in the context of a local virus infection. Interestingly, while the absolute number of CD4^+^ T cells was reduced in the CNS, which was largely due to the reduction of IFN-γ producing CD4^+^ effector T cells, the number of CTLs recruited to the CNS compartment was not significantly reduced in anti-integrin α4 treated mice (Figure 4C). Thus, integrin α4 mediated mechanisms appeared to be redundant for the recruitment of effector CD8^+^ T cells into the infected CNS.

### Th17 cells fail to clear cerebral VV infection

Similar to the murine system, neutralization of α4-integrins by natalizumab in humans mainly blocks CD4^+^ T cells from entering into the CNS compartment but spares CD8^+^ T cells [23,24]. Yet, intracerebral reconstitution of CD4^+^ effector T helper cell responses has been proposed to be necessary for efficient clearance of various viruses from the CNS including JC virus [25]. We wanted to dissect the essential features of an intracerebral T helper cell response that would be able to control viral infection. Thus, we combined a system of T cell conditional ablation of α4-integrin expression (*CD4 Cre*^*+*^*x Itga4*^*flox/flox*^ mice, α4 CKO mice) with antibody mediated depletion of CD8^+^ T cells in order to investigate the differential contribution of Th1 cells vs Th17 cells to host protection in CNS infection in the absence of CTLs. To exclude possible alterations in priming of antigen specific T helper cell responses in the peripheral immune compartment of α4 CKO mice, we compared the fractions of antigen specific T cells on day 10 after subcutaneous immunization with MVA/CFA in the spleens of *CD4 Cre*^*-*^*x Itga4*^*flox/flox*^ (wild type control) versus *CD4 Cre*^*+*^*x Itga4*^*flox/flox*^ (α4 CKO) mice (Additional file 1: Figure S1). Upon *ex vivo* restimulation with I-A^b^ restricted VV epitopes, the fractions of antigen specific (CD40L^+^) CD4^+^ T cells and their cytokine profile were similar in wild type vs α4 CKO mice. Moreover, the anti-VV neutralizing serum response was equally effective in both groups on day 10 after immunization (Additional file 1: Figure S1). Thus, sensitization for adaptive cellular immune responses against VV in draining lymph nodes and spleen was not impaired by the lack of α4-integrins on T cells.

**Figure 5:**
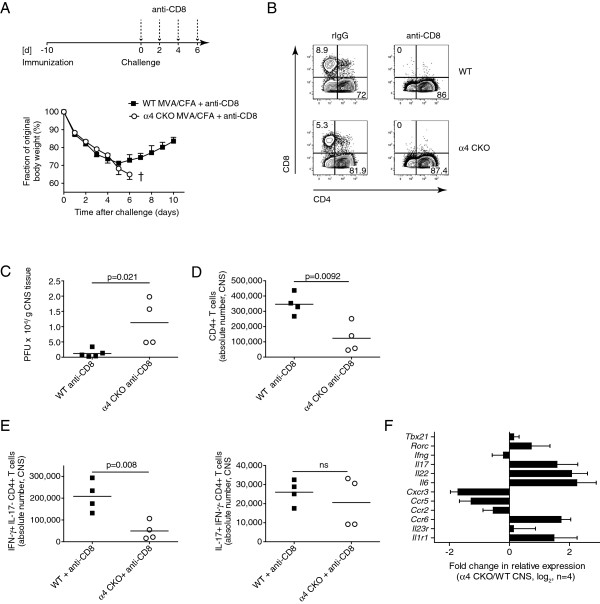
**VV specific Th17 cells are relatively enriched in the CNS compartment of α4 CKO mice but do not control i.th. VV infection.***CD4 Cre*^*-*^*x Itga4*^*flox/flox*^ mice (controls, WT) and α4 CKO mice were immunized with MVA/CFA, depleted of CD8^*+*^ T cells, and challenged i.th. with VV. **(A)** After intrathecal infection with VV, the clinical course was monitored and weight loss was calculated as percentage of initial body weight (n = 5 per group). On day 5 after infection, mice of each group were sacrificed. **(B)** The efficacy of CD8^+^ T cell depletion was analyzed by flow cytometry in CNS mononuclear cells (gate on CD3^+^CD19^-^ cells). **(C)** The viral load in the CNS was measured by means of plaque assay. CNS infiltrating CD4^+^ T cells **(D)** and absolute numbers of cytokine positive CD4^+^ T cells **(E)** were calculated by surface staining and intracellular cytokine analysis (Student’s t test, n ≥ 4 per group, representative out of n ≥ 4 independent experiments). **(F)** CD4^+^ T cells were isolated by flow cytometric purification from the CNS compartment of MVA immunized wild type vs α4 CKO mice 5 days after i.th. VV challenge. The expression profile of the indicated genes was determined by quantitative PCR analysis, n = 4.

Next, we challenged MVA immune and CD8^+^ depleted wild type and α4 CKO mice with i.th. VV. In contrast to control littermates, α4 CKO mice rapidly lost weight and succumbed to infection (Figure 5A). Even in the complete absence of CD8^+^ T cells (Figure 5B), CD4^+^ effector T helper cells were protective in wild type mice but failed to control virus replication in the CNS of α4 CKO mice (Figure 5C). Recapitulating our observations with anti-α4 integrin (PS/2) administration, α4-integrin deficient T helper cells re-isolated from the CNS of VV challenged mice exhibited a Th17 like phenotype while the number of Th1 like cells was significantly reduced in α4 CKO animals as compared with controls (Figure 5D, E). Consistent with their cytokine production upon *ex vivo* stimulation, CNS derived α4-integrin deficient T helper cells expressed higher amounts of Th17 signature markers such as *Rorc*, *Il17*, *Il22*, *Il6*, *Ccr6,* or *Il1r1* whereas Th1 associated genes like *Cxcr3*, *Ccr5,* or *Ccr2* prevailed in wild type T helper cells (Figure 5F). In conclusion, while Th1 cells appeared to be sufficient to provide host protection in intrathecal VV infection, Th17 cells failed to control cerebral virus replication in the absence of CD8^+^ T cells resulting in lethal encephalitis.

**Figure 6:**
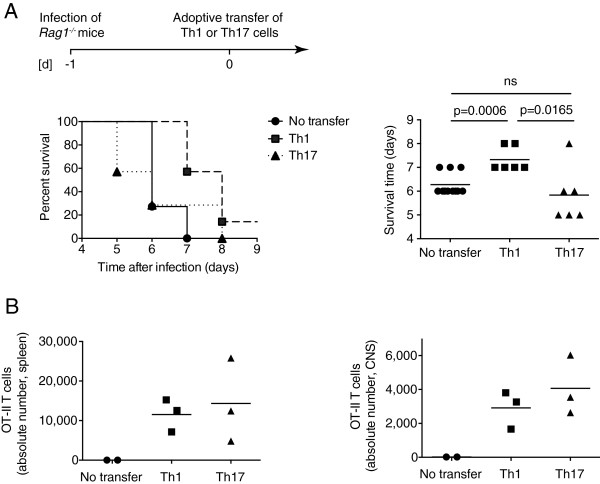
**Adoptive transfer of antigen specific Th1 cells but not Th17 cells promotes survival after intrathecal virus infection.** Naïve CD4^+^CD25^-^CD44^-^ T cells from OT-II Ova_323-339_ specific TCR transgenic mice were polarized under Th1 or Th17 conditions *in vitro*. On day four of differentiation, activated T cells were transferred into Rag1^*-/-*^ mice, which had been infected with a recombinant VV expressing ovalbumin (VV-Ova) one day earlier. **(A)** Kaplan-Meier curves of survival (left) and statistical evaluation of time to death (right) are shown. Horizontal bars in the right plot indicate mean survival time in days (ANOVA, n ≥ 5 per group). **(B)** Mice were sacrificed on day 6 after transfer and absolute numbers of adoptively transferred CD3^+^CD4^+^ T cells in spleens and CNS were assessed (means are indicated by horizontal bars, n = 3 per group).

To corroborate that Th1 immunity was sufficient to improve the outcome of intrathecal viral infection, we established a model of adoptively transferred host protection using OT-II T cells, which carry a transgenic T cell receptor specific to ovalbumin, in combination with a recombinant VV expressing ovalbumin in infected cells (VV-Ova). Naive OT-II cells were differentiated *in vitro* into Th1 cells or Th17 cells followed by transfer into *Rag1*^*-/-*^ mice, which had been infected intrathecally with VV-Ova one day prior to adoptive transfer (Figure 6). While non-transferred Rag1 deficient recipients and recipients of Th17 cells rapidly died, Th1 recipients survived significantly longer than their counterparts (Figure 6A). Despite a significant difference in clinical outcome, equal amounts of transferred Th1 cells and Th17 cells were re-isolated from the CNS of infected mice (Figure 6B). Thus, on a per cell basis, antigen specific Th1 cells appeared to be more potent in alleviating i.th. virus infection than antigen specific Th17 cells.

### Th17 cells fail to clear i.th. VV infection due to lack of perforin-1 expression

It was possible that the reduced capacity of Th17 cells to provide host protection in intrathecal VV infection was simply due to reduced availability of IFN-γ within the CNS compartment. In order to test this possibility, we neutralized IFN-γ by monoclonal antibodies in MVA immune and CD8^+^ T cell depleted wild type mice (Additional file 2: Figure S2). In this purely T helper cell dependent scenario, VV specific Th1 and Th17 cells were generated and had access to the VV challenged CNS. Notably, the mice recovered from VV encephalitis despite ablation of IFN-γ. Efficiency of IFN-γ blockade was documented by reduced levels of MHC class II expression on microglial cells in the CNS compartment (Additional file 2: Figure S2). These data suggested that IFN-γ was redundant as an effector molecule in the clearance of intrathecal VV infection and refuted the idea that diminished IFN-γ expression by Th17 cells was responsible for the failure to clear intracerebral VV infection.

**Figure 7:**
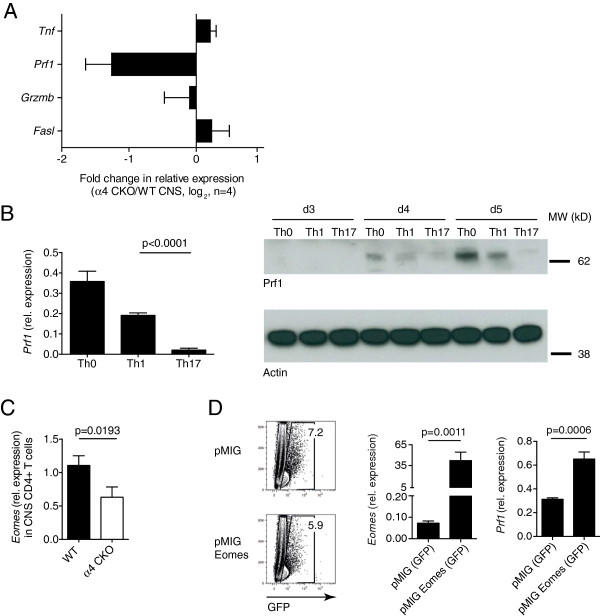
**Th17 cells fail to control VV encephalitis due to lack of Prf1 expression. (A)** CD3^+^CD4^+^ T cells were highly purified by FACS sorting from the CNS of MVA/CFA immunized wild type or α4 CKO mice on day 4 after intrathecal infection. Fold change in relative expression of the indicated genes in α4 deficient vs. wild type control CD4^+^ T cells (log scale, means + SD, n = 4). **(B)** Flow cytometrically purified naïve CD3^+^CD4^+^CD44^-^Foxp3^-^ T cells were stimulated without exogenous cytokines (Th0) or differentiated into Th1 cells (IL-12 + anti-IL-4), or Th17 cells (TGF-β + IL-6). Levels of Prf1 RNA expression were measured by quantitative RT-PCR (left in B, means + SD, Student’s t test, n = 4). Time course of Prf1 protein expression was determined by Western Blot in Th0, Th1, and Th17 cells from day 3 to day 5 of culture (right in B). **(C)** RNA levels of Eomes were measured in wild type and α4 integrin deficient T cells isolated from the CNS of VV infected mice (means + SD, n = 4). **(D)** Naïve T cells were differentiated into Th17 cells *in vitro* and transduced retrovirally with a control vector (pMIG) or an Eomes GFP vector (pMIG Eomes). GFP^+^ cells were purified by flow cytometry and RNA levels of Eomes and Prf1 were measured in control transduced Th17 cells vs Th17 cells that ectopically expressed Eomes (means + SD, n = 4).

In order to define potential molecular mechanisms of T helper cell mediated host protection in intracerebral viral infection, we screened the expression profile of highly purified CD4^+^ effector T cells isolated from the CNS of VV challenged wild type vs α4 CKO mice for molecules directly involved in virus defense (Figure 7A). CD4^+^ effector T cells expressed *Tnf*, *Grzmb*, and *Fasl* irrespective of whether they were derived from the Th1 biased inflammatory infiltrate of wild type mice or from the Th17 biased inflammatory milieu of α4 CKO mice. In contrast, the expression of perforin-1 (*Prf1*) was markedly reduced in α4-integrin deficient as compared with wild type T helper cells. In order to correlate lack of *Prf1* expression with the Th17 transcriptional program, we purified CD3^+^CD4^+^CD44^-^Foxp3^-^ naïve T cells from *Foxp3gfp*.KI mice and stimulated them without exogenous cytokines (Th0) or differentiated them into Th1 cells or Th17 cells (Figure 7B). Consistent with our *in vivo* data, we found a significant reduction in Prf1 mRNA and protein in Th17 as compared with Th1 and Th0 cells. Notably, protein expression of Prf1 was only seen at late time points (Figure 7B). Since expression of Prf1 has been reported to depend on the expression of the transcription factor eomesodermin (Eomes) [26], we measured RNA levels of Eomes in CD4^+^ T cells isolated from brains of VV infected wild type and α4 CKO mice. Consistent with the low expression of Prf1 in Th17 cells, Eomes mRNA levels were reduced in CD4^+^ T cells isolated from α4 CKO mice as compared with wild type controls (Figure 7C). To formally validate whether reduced Eomes expression in Th17 cells accounted for diminished Prf1 expression as compared with Th0 and Th1 cells, we polarized Th17 cells *in vitro* and overexpressed Eomes by retroviral transduction (Figure 7D). FACS sorted transduced (GFP^+^) Th17 cells expressed abundant levels of Eomes as compared with control vector proving effective transduction; concomitantly, Prf1 RNA was significantly increased in Eomes transduced Th17 cells. These data suggested that the failure of Th17 cells to express Prf1 was due to reduced Eomes expression.

**Figure 8:**
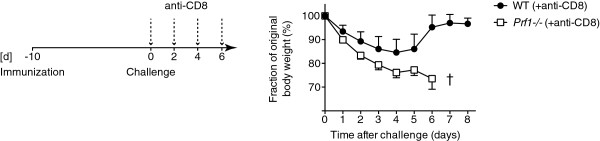
**Prf1**^**-/-**^**mice succumb to VV encephalitis.** Wild type and *Prf1*^*-/-*^ mice were immunized with MVA/CFA and continuously depleted of CD8^+^ T cells after intrathecal VV challenge. Weight courses of infected animals (n = 5 per group).

In order to explore the importance of CD4^+^ T cell derived Prf1 for successful host defense in CNS virus infection *in vivo*, we immunized Prf1 deficient mice (*Prf1*^*-/-*^) with MVA/CFA and established viral encephalitis in CD8^+^ T cell depleted animals by intrathecal infection with VV (Figure 8). While wild type mice recovered from i.th. VV challenge, Prf1 deficient mice succumbed to viral encephalitis. Numbers and fractions of CNS infiltrating immune cells were comparable between groups (data not shown) indicating that antigen specific priming in the peripheral immune compartment and establishment of inflammatory infiltrates within the CNS were not impaired in *Prf1*^*-/-*^ mice. Moreover, wild type mice that were vaccinated with MVA and then depleted of CD4^+^ T cells in addition to CD8^+^ T cells succumbed to intrathecal challenge with VV although they had similar fractions of NK cells and NK T cells in the CNS as their CD4^+^ T cell replete counterparts (Additional file 3: Figure S3) suggesting that alternative sources of Prf1 other than CD4^+^ T cells were insufficient to provide protection in this model. Taken together, these data demonstrated that Prf1 expression was indispensable for effector T helper cell mediated control of intrathecal VV infection.

## Discussion

In this study, we tested the concept that host defense against viral infections of the CNS requires distinct T helper cell subsets. We show that access inhibition to the CNS of selected T helper cell subsets by integrin targeted interventions results in the failure of host defense. Intrathecal infection with vaccinia virus (VV) was controlled in vaccinated mice when CD4^+^ and CD8^+^ T cells had access to the CNS compartment. Blockade of α4-integrins by neutralizing antibodies or T cell conditional disruption of α4-integrin expression did not prevent CTLs and Th17 cells from entering the CNS parenchyma while Th1 cells were blocked from migrating into the CNS. When CD8^+^ CTLs were depleted, host defense against intrathecal VV infection was still maintained as long as efficient Th1 like responses were operational within the CNS. In contrast, Th17 cells alone failed to rescue individuals from CNS infection despite a strong inflammatory response. Perforin-1 expression by T helper cells was required for clearance of intrathecal VV infection and Th17 cells failed to eliminate VV in the CNS due to lack of Eomes dependent Prf1 expression.

Adaptive cellular immune responses are necessary for the clearance of systemic VV infection. CD8^+^ T cells recognize epitopes from early antigens of VV but robust CD4^+^ T cell responses are also required for direct and indirect antiviral effects [27-30]. Niche restricted or organ specific infection may afford specific effector functions. For example, VV skin infection is contained and cleared by a temporally and spatially organized interaction of Ly6G^+^ innate immune cells and CD8^+^ CTLs [22]. In the ovaries, clearance of VV correlates with the number of effector memory CTLs that are present in the ovaries of VV immunized mice before challenge with the virus [31]. Here, we established an intrathecal infection model with VV and used this system as an *in vivo* surrogate to correlate the access of distinct T helper cell subsets with successful host defense. Intrathecal infection of naive wild type C57BL/6 mice with VV was lethal because adaptive antiviral immune responses did not develop upon i.th. infection alone although innate immune cells and in particular CD11b^+^CD45^high^ monocytic cells were recruited to the infected CNS. Active immunization of mice with MVA in CFA prior to i.th. VV challenge protected the animals from lethal encephalitis. Protection was independent of neutralizing antibodies. However, access of VV specific Th1 cells into the CNS compartment was necessary and sufficient for host protection.

Migration of effector cells into the CNS is mediated by chemokine receptors and integrins. Several cytolytic and non-cytolytic viral infection models of the CNS were used to investigate principles of immune cell recruitment into the CNS. Depending on the viral agent, immune cell recruitment into the CNS cleared the pathogen or in addition, caused immunopathogenic tissue damage. CCR5 and CXCR3 were identified to be important for immune cell recruitment into the CNS in west nile or MHV and LCMV infection, respectively [32-35]. Chemotaxis of immune cells must be complemented by integrin mediated interaction with endothelial cells for the transmigration of immune cells across the blood brain barrier [36]. Early work suggested that accumulation of antigen specific T cells in the CNS compartment of sindbis virus infected animals is mediated by engagement of LFA-1 but not VLA-4 [37]. Using genetic tools, we have recently shown that distinct T helper cell subsets use distinct integrins to access the CNS compartment in autoimmune inflammation. The entry of Th17 cells into the subarachnoid space most likely requires CCR6 and LFA-1 but is independent of VLA-4 [4,38]. In fact, Th17 cells express low levels of α4-integrins since TGF-β, which is required for Th17 cell commitment [17,39,40], inhibits the expression of α4-integrins [41]. Interestingly, antigen specific CD8^+^ CTLs also migrated into the CNS independently of α4-integrins (both in α4-integrin antibody blockade and T cell conditional genetic disruption of *Itga4*). This is consistent with the observation that CD4^+^ T cell are preferentially reduced in CSF of natalizumab treated patients as compared with CD8^+^ T cells resulting in the reversion of the CSF CD4^+^/CD8^+^ ratio [23]. Although one report suggested that CD8^+^ T cell influx into the CNS was prevented by a blocking antibody to α4-integrins in an intracerebral corona virus infection model, it is unlikely that the recruitment of antigen specific CTLs was assessed by Ifergan et al. since their model did not allow for active priming and activation of adaptive T cell responses in the systemic lymphoid compartment prior to intracerebral challenge [42]. In contrast to Th17 cells, VLA-4 expression is indispensable and non-redundant for the capacity of antigen experienced Th1 cells to infiltrate into the subarachnoid space and the CNS compartment. Here, we provide evidence that MHC class II positive infected phagocytic cells in the CNS need to be cleared by Th1 cells in order to rescue mice from lethal encephalitis. Thus, MVA-immune T cell conditional α4 KO mice succumbed to intrathecal VV challenge because they failed to recruit Th1 cells into the CNS despite regular Th17 cell accumulation.

Antigen specific CD4^+^ T cells communicated with virus infected macrophages within the CNS parenchyma and only eliminated the infected cells when they were sufficient in perforin-1 expression. In ectromelia infection, a natural mouse pox infection, perforin-1 expression by CD4^+^ T cells is required to clear the pathogen, and CD4^+^ T cell mediated killing of hematopoietic MHC class II expressing infected target cells occurs in the draining lymph nodes, the liver, and the spleen [28]. Direct cytotoxic effects of antigen specific CD4^+^ T cells were also reported in systemic VV infection [43]. In our model, T cell conditional α4-integrin deficient mice behaved like perforin-1 deficient animals and succumbed to infection because perforin-1 expressing Th1 cells did not reach the CNS compartment in the absence of VLA-4. Like in EAE, the cytokine phenotype of T helper cells that entered into the CNS compartment in the absence of α4-integrin showed a Th17 profile [4]. Th17 cells did not express perforin-1. Initial Th17 cell commitment is sensitive to IL-2 and high IL-2 concentrations inhibit Th17 differentiation [44]. In contrast, IL-2 promotes the expression of granzyme B and perforin-1 and boosts cytotoxic capacity of CD4^+^ T cells [45]. While the transcriptional control of granzyme B and perforin-1 has in part been investigated in CD8^+^ T cells [46], we found that perforin-1 expression in Th1 cells is associated with Eomes and overexpression of Eomes in Th17 cells partially reconstituted perforin-1 expression. Similar observations have been reported for Th2 cells that *per se* also lack perforin-1 [26], which correlates with their low cytotoxic efficacy. However, lack of perforin-1 expression in Th17 cells may not be the only mechanism to impair their antiviral capacity. For example, Th17 cells are able to mount a protective response in primary lung infection with influenza in IL-10 KO mice suggesting that Th17 cells are particularly susceptible to IL-10 mediated suppression [47]. In addition, induction of prosurvival factors by Th17 cells may contribute to viral persistence in the CNS in Theiler’s virus infection [48]. Conversely, it is possible that in some anatomical niches, Th17 cells may contribute to protective antiviral immune responses, e.g. by effective IL-17 mediated recruitment of neutrophils in lung infection [49]. Also, IL-21, which is produced by Th17 cells, might have a role in the modulation of primary and recall CTL responses by increasing their proliferation and reducing their sensitivity to exhaustion [50]. However, overall it is a more common scenario that antiviral Th17 responses fail to clear viral pathogens but rather drive deleterious immunopathology in the skin [51], in the cornea [52], and also in the CNS.

We have shown that blockade or genetic lack of α4-integrins on T cells selectively modulated the access of T helper cell subsets to the CNS. Preferential limitation of Th1 trafficking jeopardized host defense against intrathecal VV infection. Nevertheless, our experimental system has several limitations: First, i.th. inoculation of VV does not represent a natural route of CNS infection. Yet, immunization with virus antigens in CFA provides an efficient way to generate both an antigen specific Th1 and Th17 response in the secondary lymphoid tissue and to study their trafficking and effector functions upon i.th. challenge with a well characterized pathogen. Second, i.th. VV infection results in an acute encephalomyelitis, which causes rapid decay and death of infected animals; in contrast, JC virus induced PML is due to insufficient control of a latent virus infection of oligodendrocytes. However, the intracerebral anti-JC virus CD4^+^ T cell response contributes substantially to JCV control because efficient clearance of JC virus from the CNS after immune reconstitution by wash-out of natalizumab in MS patients with PML is associated with the re-occurrence of Th1 cells in the CNS [53].

## Conclusions

Our experimental model provides a mechanistic explanation for insufficient virus control under conditions of altered T cell migration. Our data raise the concern that treatment of humans with compounds like natalizumab that interfere with T cell trafficking not only by quantitatively reducing the migration of T cells into the CNS but also by selectively targeting distinct T cell subsets may evoke serious deficiencies in cellular immune responses at privileged anatomical sites like the CNS. As a result, primary infections or unexpected reactivations of latent viruses may be facilitated.

## Supporting data

The data sets supporting the results of this article are included within the article and its additional files.

## Competing interests

The authors declare that they have no competing interests.

## Authors’ contributions

TK, VR and AM designed the study and wrote the manuscript. VR and AM contributed equally, carried out all the experiments and where herein supported by GG, FP, and SH. DHB provided fluorescence-labeled MHC class I H-2K^b^/B8R_20-27_ (TSYKFESV) multimers. Histological analyses were carried out with the support of MH’s group. GG, DHB, MH, BH and ID gave conceptional input and revised the manuscript. All authors read and approved the final manuscript.

## Supplementary Material

Additional file 1: Figure S1 α4 integrin deficiency in T cells does not alter priming or humoral responses against VV after immunization. (A) *CD4 Cre*^*-*^*x Itga4*^*flox/flox*^ mice (wild type control (WT)) or *CD4 Cre*^*+*^*x Itga4*^*flox/flox*^ mice (α4 CKO) were immunized with MVA/CFA. On day 10 after immunization, splenocytes were isolated and restimulated with an I-Ab restricted peptide epitope mix of VV. Fractions of antigen specific cytokine producing CD4^+^ T cells were determined by intracellular CD40L (CD154) and cytokine staining. Numbers in the gates represent means ± SD, n = 3. (B) Specific serum neutralization capacity of sera collected on day 10 after immunization of wild type or α4 CKO mice vaccinated with PBS/CFA or MVA/CFA (specific neutralization capacity in percent of maximum, means + SD, n = 5). Click here for file

Additional file 2: Figure S2 IFN-γ expression in CD4^+^ T cells is not required for virus control in the CNS. (A) Wild type mice were immunized with MVA/CFA and challenged intrathecally with VV on day 10. Starting one day before VV challenge, neutralizing antibodies to IFN-γ (or control IgG) and depleting antibodies to CD8 were applied alternatingly every other day. Weight courses of infected animals were measured every day after infection and depicted relative to initial body weight (n = 5 per group). (B) Expression of MHC class II on CNS microglial cells (gating on CD11b^int^CD45^int^, upper panel) was measured on day 6 after infection using surface staining and flow cytometric analysis. Numbers indicate percentages. Histogram: black line: MHC class II expression on migroglia from control IgG treated mice, grey/shaded line: MHC class II expression on microglia from anti-IFN-γ treated mice. The numbers in the histogram represent mean fluorescence intensities. Representative out of 3 independent experiments. Click here for file

Additional file 3: Figure S3 CD4^+^ T cells mediate protection against VV encephalitis in the absence of CD8^+^ T cells. Wild type mice immunized with MVA/CFA were challenged i.th. with VV and depleted of CD8^+^ T cells. Concomitantly, animals were treated with isotype control or depleting antibodies to CD4 every other day. (A) Percentage of surviving mice are depicted in Kaplan-Meier curves of survival (n = 6 per group). (B) CNS infiltrating mononuclear cells were analyzed by flow cytometry. Upper panel: Staining for CD4 and CD8 in the CD3^+^ T cell gate. Lower panel: Staining for CD3 and Nk1.1 in the CD4^-^ gate (representative out of 4 independent experiments). Click here for file
